# Effectiveness of Phone Call Follow-Ups in Improving Patient Compliance to Post-extraction Instructions: A Cross-Sectional Study

**DOI:** 10.7759/cureus.31499

**Published:** 2022-11-14

**Authors:** Sundar Ramalingam, Obaid Alotaibi, Ziyad Alqudairy, Abdulrahman Alnutaifi, Abdulaziz Alotaibi

**Affiliations:** 1 Oral and Maxillofacial Surgery, King Saud University College of Dentistry, Riyadh, SAU; 2 College of Dentistry, King Saud University, Riyadh, SAU

**Keywords:** postoperative complications, patient education, phone call follow-up, patient compliance, post-extraction instructions

## Abstract

Background

Dental extraction is a commonly performed oral surgical procedure. The manner in which post-extraction instructions are given to patients may impact their understanding and adherence to instructions. This study aimed to evaluate the effectiveness of phone call follow-ups over conventional verbal and written post-extraction instructions in terms of patient compliance in Riyadh, Saudi Arabia.

Methodology

After obtaining informed consent, patients undergoing dental extraction at the Department of Oral and Maxillofacial Surgery were randomly enrolled into one of the three groups based on the mode by which post-extraction instructions were administered. Group A received verbal and written instructions only, and Group B and Group C received additional phone call follow-up on the first postoperative day and the first and third postoperative days, respectively. After seven days, all patients answered a questionnaire to quantify the level of compliance on a score out of 10, which was the outcome variable. The nature of receiving post-extraction instruction was the primary predictor variable. Age, gender, and type of extraction (surgical or non-surgical) were secondary predictors. Descriptive statistical analysis and statistical comparison of mean compliance scores between the groups and the effect of interaction between primary and secondary predictors on the outcome variable were carried out at a 95% significance level (p < 0.05).

Results

A total of 135 patients (75 males and 60 females; mean age = 36.2 years) were included in the study. While the overall mean compliance score was 8.36 ± 1.08, Group C (9.14 ± 0.78, n = 42) had a significantly higher level of compliance than Group B (8.48 ± 1.01, n = 40) and Group A (7.64 ± 0.83, n = 53) (one-way analysis of variance (ANOVA) F = 34.937; p < 0.001). Similarly, the level of compliance in Group B was significantly higher than that in Group A (p < 0.01). There was no significant statistical relationship between the secondary predictors and the compliance scores. Similarly, two-way ANOVA revealed no statistically significant effect of interaction between the primary and secondary predictor variables on the compliance scores. Individually, the least complied instruction, across all groups, was “rinsing with saline once every six hours for four days,” and “biting on a gauze pack for 30 minutes” was the most complied instruction.

Conclusions

Phone call follow-up after teeth extraction improves patient compliance with post-extraction instructions through reinforcement and education. While a single phone call follow-up one day after extraction along with verbal and written instructions increased patient compliance significantly, a second phone call follow-up on the third postoperative day yielded the best level of compliance.

## Introduction

Tooth extractions remain among the most frequently performed dental procedures and the oldest oral surgical procedure [1]. The patient’s quality of life, including their ability to speak, eat, and feel, may be compromised by this procedure. While complications during tooth extraction are minimally expected, post-extraction complications such as pain, swelling, trismus, dry socket, ulcers, paresthesia, and bleeding frequently occur [2]. Fortunately, most complications following dental extractions can be avoided by following proper clinical procedures and complying with precise postoperative instructions. Researchers have repeatedly emphasized the need for and importance of patient education and compliance following a tooth extraction to reduce health burden and improve patient quality of life [3]. Non-compliant patients have been reported to have an exponentially higher incidence of dry socket (57.1%) post-extraction [4], thereby highlighting the importance of understanding and complying with postoperative instructions.

In general, when postoperative instructions are correctly followed, patients recover more rapidly, experience minimal to no morbidity, and have better postoperative quality of life [5]. Alexander et al. reported that most routinely available written postoperative instructions in dentistry are riddled with poor wording and terminology which can interfere with adherence to postoperative recommendations [6]. Based on a pilot study, it has been shown that patient education, oral health awareness, and treatment satisfaction are the main variables that positively affect patient compliance after periodontal surgery [7]. On the other hand, anxiety or stress makes it difficult for patients to focus on the information provided by the surgeon after a surgical procedure [5]. Additionally, the inability to read written information about the management of their disease, and not receiving information about the medication prescribed, have been shown to negatively influence adherence to therapeutic regimens [8]. While the doctor-patient rapport had a great positive impact on compliance with treatment and post-treatment recommendations, demographic characteristics such as treatment geography, place of residence, and ethnicity did not affect compliance significantly [9].

Considering that post-extraction instructions are aimed at reducing postoperative sequelae, there has always been advocacy toward the use of alternative forms of delivering them to increase levels of adherence and compliance [4,7]. In a study conducted in 1992, Aloy-Prosper et al. concluded that phone call follow-up increases the level of compliance significantly [5]. Zheng et al. (2020) reported the beneficial effects of a postoperative online follow-up along with written instructions, wherein there was an overall improvement in patient quality of life post-dental extraction [10]. Vallerand et al. showed that, in patients undergoing third molar extractions, postoperative pain control and satisfaction were greater for patients who receive detailed postoperative information and recommendations thereafter [11].

Although administering post-extraction instructions through verbal and printed means in Arabic (local language) and English form part of good clinical practice protocol in Saudi Arabia [12], information regarding the reinforcement of the instructions through alternative means is not available within published literature. Therefore, the present study was conducted under the premise that providing a phone call follow-up to previously administered verbal and printed post-extraction instruction would enhance adherence and compliance. This study aimed to evaluate the effectiveness of phone call follow-up over conventional verbal and written post-extraction instructions in terms of patient compliance in Riyadh, Saudi Arabia.

## Materials and methods

Study design

From January 2022 to September 2022, a cross-sectional study was conducted among patients who had any tooth extracted in the Oral and Maxillofacial Surgery Department of King Saud University, Riyadh, Saudi Arabia. The study was conducted in accordance with the Declaration of Helsinki guidelines for human research [13]. Before participating in the study, all patients were given information about the study and asked to sign a written informed consent form. The study design was approved by the Institutional Review Board (IRB) of King Saud University (approval number: E-22-6719).

Selection criteria

Adult Saudi patients above 18 years of age undergoing extraction and agreeing to participate in the study by signing an informed consent form were eligible for inclusion. Patients with mental illnesses or psychiatric disorders which rendered them unable to understand and follow postoperative instructions independently were excluded. All participating patients were randomly assigned using an open-source computer program (Random Sequence Generator, https://www.random.org/) to one of the following three study groups based on how the postoperative instructions were administered and the phone call follow-up was reinforced: Group A - verbal and printed instructions only, as described in Table 1; Group B - verbal and printed instructions, as described in Table 1, and a phone call follow-up to reinforce instructions on the first postoperative day; Group C - verbal and printed instructions, as described in Table 1, and a phone call follow-up to reinforce instructions on the first and third postoperative days.

**Table 1:** Printed post-extraction instructions provided after the extraction.

Post-extraction instructions
Prepare a hot saline mouthwash by adding one teaspoonful of salt to a tumbler of hot but not scalding water. Bathe the mouth gently with this at least four times daily for four days, starting at bedtime on the day of the extraction – not before
It is important that a blood clot develops in the socket. Do not touch or poke the socket with the tongue or finger. If the dentist gives you a cotton or gauze pad to bite on when leaving the clinic, throw it away (into a proper waste receptacle) after 20 minutes. Its sole purpose is to keep your mouth comfortable for a few minutes while a blood clot forms
A little blood-stained saliva is a normal consequence of tooth extraction and of no importance. However, if bleeding persists for more than one or two hours, hold a small pad of clean cloth over the socket with gentle pressure for 10 minutes from a finger or from the opposing teeth. If bleeding is very severe or persists for more than eight hours, contact the dentist again
Eat soft foods such as minced meat, ice cream, and milk pudding for 48 hours after the operation. Keep food on the side of the mouth opposite to the extraction area
If you are given antibiotic tablets or capsules and analgesics complete the full course, as prescribed by the dentist. Do not stop when you feel better
Be careful to avoid biting or scalding the area made numb by local (injection) anesthesia

The standard set of post-extraction instructions was obtained from the Department of Oral and Maxillofacial Surgery specialized clinics and was provided in the local language (Arabic) along with a translated version in English. None of the patients participating in the study received any preferential treatment or financial rewards. All study participants and clinicians performing the extraction were blinded to the random patient grouping, which was only known to the authors of this study.

Procedure and data collection

The extraction was performed following standardized oral surgery clinical practice guidelines relating to anamnesis, aseptic protocol, pain control, and postoperative therapeutic management. At the time of the extraction appointment, demographic information such as age, gender, tooth/teeth to be extracted, and type of extraction (surgical or non-surgical) were recorded. Seven days post-extraction, patients were recalled to answer a questionnaire (Table 2), which quantitatively assessed their level of compliance with post-extraction instructions. The primary predictive variable was how the post-extraction instructions were administered and the presence or absence of phone call follow-up at varying intervals, as per the groups previously mentioned. Age, gender, and type of extraction were identified as secondary predictors. Patient age was further stratified into age groups (young adults - 18 to 30 years; middle-aged - 31 to 45 years; older adults - 46 to 60 years; and senior citizens - 61 years and above). The key outcome variable of interest was the level of compliance score (quantified out of 10) according to the validated questionnaire shown in Table 2.

**Table 2:** Questionnaire to assess the level of compliance with post-extraction instructions.

Items	Yes (1 point)	No (0 point)
Did you keep pressing a cotton or gauze pad for 20 minutes after extraction?		
Did you avoid disturbing the clot with your tongue or finger?		
Did you avoid vigorous rinsing or spitting during the postoperative period?		
Did you rinse your mouth gently with warm saline four times daily for four days?		
Did you take soft foods such as minced meat, milk pudding, etc. for 48 hours after extraction?		
Did you maintain proper oral hygiene?		
Did you avoid smoking after the extraction period?		
Did you avoid biting or scalding the area made numb by local anesthesia?		
Did you complete the full course of antibiotics prescribed by the dentist? (If it were prescribed)		
Did you take analgesics and anti-inflammatory medication according to the recommendations of the surgeon?		
Total score (Sum of the scores above)		

Statistical analysis

The collected data were tabulated and statistically evaluated using SPSS version 20 (IBM Corp., Armonk, NY, USA). Descriptive statistics were done for all variables. Statistical differences between the different patient groups were analyzed using parametric tests for all quantitative variables. While the compliance score was compared to the age of participants using Pearson’s correlation, an independent t-test was used to compare the scores with gender and type of extraction. Additionally, one-way analysis of variance (ANOVA) with post-hoc analysis was used to compare the compliance scores between different age groups and the level of compliance between the three study groups. To study the effect of interaction between the primary and secondary predictor variables (age group, gender, and type of extraction) on the compliance score, a two-way ANOVA was done. All statistical tests were performed assuming at least a 95% level of significance (p < 0.05).

## Results

The final study sample included 135 patients (75 males and 60 females), with a mean age of 36.2 years (range = 18-73 years). While Group A had 53 patients (mean age = 35.3 years, range = 18-66 years), Group B had 40 patients (mean age = 36.6 years, range = 18-73 years), and Group C had 42 patients (mean age = 36.9 years, range = 18-67 years). The sample size distribution across each group, gender, type of extraction, and age group is shown in Table 3.

**Table 3:** Descriptive statistics across all study groups and demographic variables.

Descriptive data	Study groups	Total study population	Gender		Type of extraction		Age group			
			Male	Female	Surgical	Non-surgical	Young adults (18–30 years)	Middle-aged (31–45 years)	Older adults (46–60 years)	Senior citizens (>60 years)
Sample size (n) and distribution	Overall	135	75	60	50	85	51	58	18	8
	Group A	53	23	30	15	38	18	26	7	2
	Group B	40	24	16	12	28	16	16	4	4
	Group C	42	28	14	23	19	17	16	7	2
Compliance score (mean ± SD)	Overall	8.36 ± 1.08	8.36 ± 1.10	8.35 ± 1.06	8.16 ± 1.25	8.47 ± 0.95	8.41 ± 1.08	8.38 ± 1.09	8.44 ± 0.98	7.62 ±1.08
	Group A	7.64 ± 0.83	7.61 ± 0.94	7.67 ± 0.76	7.01 ± 0.85	7.89 ± 0.69	7.56 ± 0.86	7.73 ± 0.87	7.57 ± 0.79	7.51 ± 0.71
	Group B	8.48 ± 1.01	8.29 ± 1.08	8.75 ± 0.86	7.92 ± 0.99	8.71 ± 0.94	8.62 ± 1.03	8.51 ± 0.97	9.01 ± 0.88	7.25 ± 0.90
	Group C	9.14 ± 0.78	9.04 ± 0.79	9.36 ± 0.75	9.04 ± 0.88	9.26 ± 0.65	9.12 ± 0.70	9.31 ± 0.79	9.03 ± 0.58	8.52 ± 1.12

The overall mean level of compliance score was 8.36 ± 1.08. Patients in Group C (n = 42) who received verbal and written post-extraction instructions and phone call follow-up on the first and third postoperative days had the highest level of compliance with instructions (9.14 ± 0.78). This was followed by patients in Group B (n = 40) who received only one phone call follow-up on the first postoperative day (8.48 ± 1.01). The least level of compliance was observed among patients in Group A (n = 53, compliance score = 7.64 ± 0.83) who received only verbal and written instructions and no phone call follow-up. Based on one-way ANOVA, the difference in the level of compliance among the three groups was statistically significant (F = 34.937, p < 0.001). Post-hoc analysis revealed a significantly higher mean compliance score in Group C than in Groups A and B (p < 0.01). Similarly, group B had a significantly greater mean compliance score than Group A (p < 0.01). Descriptive statistics for compliance score across each group, gender, type of extraction, and age group are shown in Table 3.

**Figure 1:**
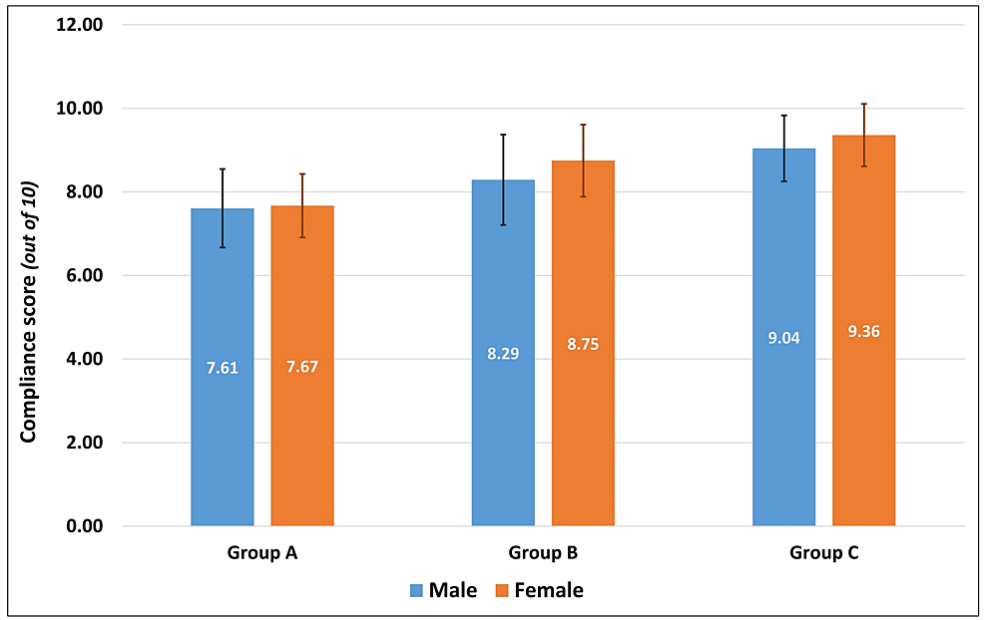
Comparison of compliance scores between male and female patients in the three study groups.

**Figure 2:**
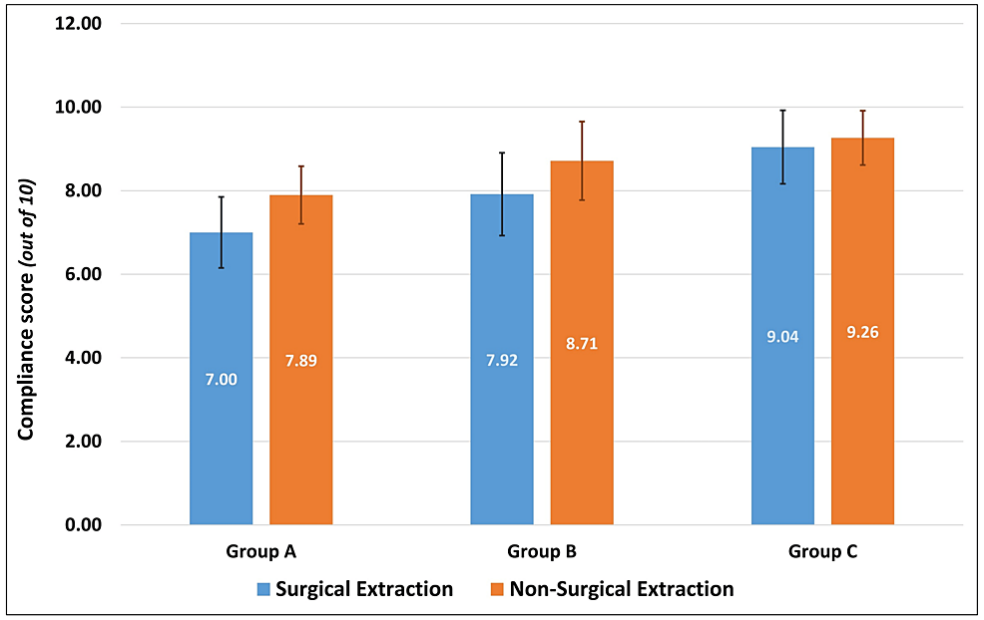
Comparison of compliance scores between surgical and non-surgical extraction patients in the three study groups.

**Figure 3:**
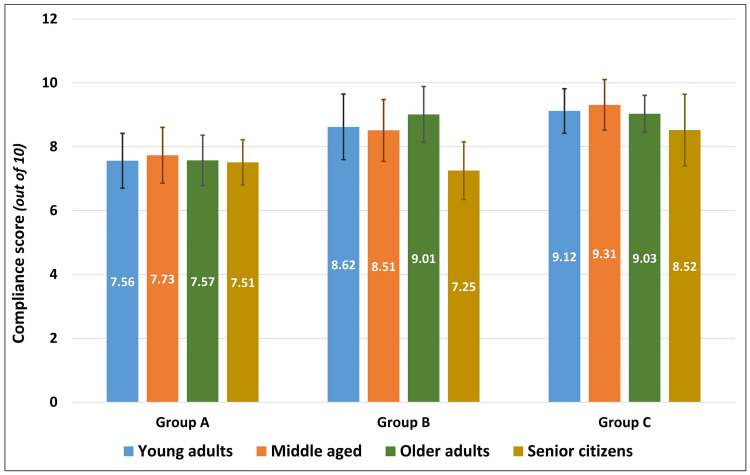
Comparison of compliance scores between young adults, middle-aged, older adults, and senior citizens in the three study groups.

Correlating the age of the patients to their respective levels of compliance scores, there was a statistically insignificant negative correlation (Pearson’s r = -0.091, p = 0.292). Independent t-test to compare the level of compliance between patients based on their gender (t = 0.053, df = 133, p = 0.957) and type of extraction (t = -1.631, df = 133, p = 0.105) showed no statistically significant difference based on the secondary predictor variables. Similarly, one-way ANOVA to identify differences in the compliance scores between participants of different age groups was not statistically significant (F = 1.338, df = 134, p = 0.265). While the overall compliance scores were almost similar between males and females, within each group, female patients indicated greater levels of compliance than male patients. Patients who underwent a non-surgical extraction complied better with post-extraction instructions than those who had a surgical extraction, which was also similarly seen in the individual groups. While the compliance scores were almost similar among young adults, middle-aged, and older adults, across all the study groups, senior citizens had poor compliance scores than the rest. Despite stratification based on gender, type of extraction, and age groups, there was a general increase in the mean level of compliance from Group A to Group B and to Group C, across all strata (Table 3). Two-way ANOVA to identify the effect of statistical interaction between the study group and gender (F = 0.619, p = 0.540), study group and type of extraction (F = 2.036, p = 0.135), and study group and age group (F = 0.776, p = 0.590) on the compliance scores showed no statistically significant differences (Figures 1-3).

Regarding following the instructions given, there was a reasonable to good degree of compliance for all instructions except “Avoiding vigorous rinsing/spitting for one day,” “Rinsing with salt and warm water once every six hours for four days,” and “Proper oral hygiene” (Table 4). Among the above three, “rinsing with warm saline” was the least complied instruction in all three groups. Interestingly, for all the aforementioned instructions wherein poor compliance was noted, the percentage of compliers increased with increasing follow-up through phone calls, i.e., Group C was better than Group B and Group B was better than Group A. The degree of compliance and their respective levels of significance (p-values) for each questionnaire item based on the chi-square test are shown in Table 4.

**Table 4:** Percentage compliance and the level of significance (p-value) for each post-extraction instruction in the three study groups.

Post-extraction instruction	Group A	Group B	Group C	P-value
Pressing/biting on gauze for 20 minutes	100.0%	97.5%	100.0%	0.302
Avoiding disturbance of the clot	94.3%	100.0%	100.0%	0.093
Avoiding vigorous rinsing/spitting for one day	52.8%	72.5%	85.7%	0.002
Rinsing with salt and warm water once every six hours for four days	9.4%	45.0%	61.9%	<0.001
Soft diet for 48 hours after the extraction	88.7%	97.5%	92.9%	0.274
Proper oral hygiene	39.6%	57.5%	81.0%	<0.001
Avoid smoking	86.8%	87.5%	97.6%	0.158
Avoid biting the anesthetized area	96.2%	97.5%	100.0%	0.459
Complete the course of antibiotics as advised	98.1%	95.0%	97.6%	0.657
Complete the course of analgesics as advised	98.1%	97.5%	97.6%	0.977

## Discussion

Patient compliance with postoperative instructions is a very important aspect of health-related quality of life outcomes. Tooth extraction frequently impacts the patient’s ability to eat, speak, and feel due to the procedure’s requirement for gingival and mucoperiosteal flap elevation, bone removal, and tooth sectioning. These surgical procedures may eventually cause pain, swelling, and bleeding, and significantly lower patients’ quality of life [14]. The importance of following post-extraction instructions correctly leads to patients recovering rapidly after oral surgery, thereby reducing morbidity [4]. The way a professional communicates postoperative care instructions (verbally and/or in writing) may affect how well patients comprehend and follow them, which can interfere with their ability to recover from surgery [2]. Some studies have demonstrated that written instructions are a beneficial adjunct to verbal instructions for increasing patient understanding [11]. Similar studies have found that using alternative methods to communicate post-extraction instructions, such as through calls and text messages, improved compliance [4]. Meanwhile, a clinical investigation published by Alvira-González and Gay-Escoda found no variations in the degree of compliance to postoperative instructions based on how they were delivered to the patient (verbal, written, and a group that received written additional information [15].

There are no studies reported in the literature from our geographic region about the influence of a phone call follow-up upon adherence to instructions provided to patients. To our knowledge, this is the first study of its kind conducted in the Saudi population that examines the benefit of various ways of phone call follow-up to increase compliance with postoperative instructions in the field of dentistry. Accordingly, we surmised that phone call follow-up during the recovery period would improve the implementation of instructions and answer any questions or worries that may have arisen following tooth extraction. Therefore, the main goal of this study was to compare the effectiveness of including different periods of phone call follow-up along with traditional verbal and written post-extraction instructions, as well as its effect on compliance with the instructions in Riyadh, Saudi Arabia.

It was observed in the present study that women were slightly more compliant with post-extraction instructions than men. Males are more likely to neglect their oral health and have poorer oral hygiene habits than females, due to a combination of biological, societal, and gender-related factors. Hence, they often report greater incidences of periodontal disease, oral pathologies, and dental trauma [16]. Additionally, males, in general, are more likely to use any type of tobacco product compared to females [17], which might explain the reason why females were more compliant with post-extraction smoking cessation, as seen in this study. The type of extraction also plays a role in patient compliance. In this study, patients who underwent non-surgical tooth extraction had greater compliance scores compared to patients who underwent surgical extraction, which may be explained due to postoperative anxiety or stress that makes it difficult for patients to focus on the information the surgeon provides [18]. Increasing age has always been considered a detriment to the degree of compliance with therapeutic and postoperative instructions [9]. In the present study, it was observed that senior citizens above the age of 60 years had poor overall compliance scores than young adults, middle-aged, and older adults. Although not statistically significant, in this study, phone call follow-up resulted in increased compliance among participants of all age groups, including senior citizens. Hence, the benefit of a phone call follow-up is that it will increase patient adherence to post-extraction instructions by not only acting as a reminder and positive reinforcement tool but also by helping resolve patient queries relating to pain and bleeding after extraction [10]. It has further been observed that a phone call follow-up reduces the frequency of patients returning to the hospital due to postoperative sequelae [5].

In the present study, certain post-extraction instructions witnessed lower levels of compliance from the patients compared to the other instructions. For instance, “rinsing the mouth with warm saline once in six hours,” “following proper oral hygiene,” and “avoiding vigorous rinsing/spitting for one day” were poorly complied with by patients in all three groups, and the difference was statistically significant. However, there was a noticeable improvement in adhering to the above instructions with increasing frequency of phone call follow-up, thereby indicating the reinforcing role of postoperative phone calls. The most likely reason behind patient negligence toward “regular mouth rinsing with saline after extraction” was primarily attributed to the alleviation of pain symptoms, which let patients return to their regular lives without worrying about it. Similarly, the possible reason for “avoiding rinsing/spitting” and “proper oral hygiene” stemmed from ignorance about how to clean the oral cavity adjoining the extracted site. The evidence that phone call follow-up improved adherence to the above instructions is proof enough of their educative role along with verbal and written post-extraction instructions. The fact that a clinician could educate his/her patient about postoperative instructions and recommendations is imperative for building clinician-patient trust and relationship [4].

One limitation of the present study was that it was conducted only at a single center in Riyadh, Saudi Arabia, and its findings can be extrapolated only to the local geographical region. This necessitates further multicenter studies across different geographical regions to further understand the role of phone call follow-up in improving compliance with post-extraction instructions.

## Conclusions

Based on the present study and its outcomes, it can be safely concluded that phone call follow-up after teeth extraction helps patients improve their compliance with post-extraction instructions through reinforcement and education. Furthermore, phone call follow-up results in enhanced compliance to often neglected post-extraction instructions such as “rinsing with saline,” “avoiding vigorous rinsing/spitting,” and “the importance of proper oral hygiene.” While a single phone call follow-up one day after the extraction, along with verbal and written instructions, increased patient compliance significantly, a second phone call follow-up on the third postoperative day yielded the best results regarding the level of compliance scores. Therefore, follow-up communication through a simple phone call at spaced-out intervals should be included in the post-extraction instruction regimen to improve patient compliance and quality of life outcomes.

## References

[1] Abhinav RP, Selvarasu K, Maheswari GU, Taltia AA (2019). The patterns and etiology of maxillofacial trauma in South India. Ann Maxillofac Surg.

[2] Saravanan K, Santhosh Kumar MP (2021). Assessment of post extraction complications in Indians. Bioinformation.

[3] van Wijk AJ, Buchanan H, Coulson N, Hoogstraten J (2010). Preparatory information for third molar extraction: does preference for information and behavioral involvement matter?. Patient Educ Couns.

[4] Alsaleh MK, Alajlan SS, Alateeq NF, Alamer NS, Alhobeira HA, Khan S (2018). Alveolar osteitis: patient's compliance with post-extraction instructions following permanent teeth extraction. J Contemporary Dent Pract.

[5] Aloy-Prósper A, Pellicer-Chover H, Balaguer-Martínez J, Llamas-Monteagudo O, Peñarrocha-Diago M (2020). Patient compliance to postoperative instructions after third molar surgery comparing traditional verbally and written form versus the effect of a postoperative phone call follow-up a: a randomized clinical study. J Clin Exp Dent.

[6] Alexander RE (1999). Patient understanding of postsurgical instruction forms. Oral Surg Oral Med Oral Pathol Oral Radiol Endod.

[7] Shah R, Thomas R, Bhandari S, Mehta DS (2017). Influence of various factors on patient compliance after periodontal therapy: a pilot study. J Indian Soc Periodontol.

[8] Parra DI, Romero Guevara SL, Rojas LZ (2019). Influential factors in adherence to the therapeutic regime in hypertension and diabetes. Invest Educ Enferm.

[9] Mirzakouchaki B, Shirazi S, Sharghi R, Shirazi S (2016). Assessment of factors affecting adolescent patients' compliance with Hawley and vacuum formed retainers. J Clin Diagn Res.

[10] Zheng X, Zhao J, Wang Z (2021). Postoperative online follow-up improves the quality of life of patients who undergo extraction of impacted madibular third molars: a randomized controlled trial. Clin Oral Investig.

[11] Vallerand WP, Vallerand AH, Heft M (1994). The effects of postoperative preparatory information on the clinical course following third molar extraction. J Oral Maxillofac Surg.

[12] Yaser A, Ahmad MAJ, Badr MF (2020). Can knowledge about post extraction instruction affect post extraction behavior: a cross sectional study among the patients visited Taibah University College of Dentistry Clinic, Madinah, KSA. IMSEAR.

[13] Goodyear MD, Krleza-Jeric K, Lemmens T (2007). The Declaration of Helsinki. BMJ.

[14] McGrath C, Comfort MB, Lo EC, Luo Y (2003). Changes in life quality following third molar surgery--the immediate postoperative period. Br Dent J.

[15] Alvira-González J, Gay-Escoda C (2015). Compliance of postoperative instructions following the surgical extraction of impacted lower third molars: a randomized clinical trial. Med Oral Patol Oral Cir Bucal.

[16] Lipsky MS, Su S, Crespo CJ, Hung M (2021). Men and oral health: a review of sex and gender differences. Am J Mens Health.

[17] Bassiony MM (2009). Smoking in Saudi Arabia. Saudi Med J.

[18] Schouten BC, Eijkman MA, Hoogstraten J (2003). Dentists' and patients' communicative behaviour and their satisfaction with the dental encounter. Community Dent Health.

